# Renal Vascular Response to Angiotensin II Administration in Two Kidneys-One Clip Hypertensive Rats Treated with High Dose of Estradiol: The Role of Mas Receptor

**DOI:** 10.1155/2021/6643485

**Published:** 2021-03-01

**Authors:** Samira Choopani, Mehdi Nematbakhsh

**Affiliations:** ^1^Water and Electrolytes Research Center, Isfahan University of Medical Sciences, Isfahan, Iran; ^2^Department of Physiology, Isfahan University of Medical Sciences, Isfahan, Iran; ^3^Isfahan ^MN^ Institute of Basic and Applied Sciences Research, Isfahan, Iran

## Abstract

**Backgrounds:**

High blood pressure is one of the most important causes of death around the world. The renin-angiotensin system (RAS) and estradiol are two important items that regulate arterial blood pressure in women. However, hypertension, RAS, and sex hormone estradiol may influence renal vascular responses. This study was designed to determine the role of Mas receptor (MasR) on renal vascular response to angiotensin II (Ang II) administration in two kidneys-one clip (2K1C) hypertensive rats treated with estradiol.

**Method:**

The ovariectomized rats were subjected to 2K1C or non-2K1C and simultaneously treated with estradiol (500 *μ*g/kg/weekly) or placebo for a period of 4 weeks. Subsequently, under anesthesia, renal vascular responses to graded doses of Ang II administration with MasR blockade (A779) or its vehicle were determined.

**Results:**

A779 or its vehicle did not alter mean arterial pressure (MAP), renal perfusion pressure (RPP), and renal blood flow (RBF). However, in non-2K1C rats, Ang II infusion decreased RBF and increased renal vascular resistance (RVR) responses in a dose-related manner (*P*treat < 0.0001). The greatest responses were found in ovariectomized estradiol-treated rats that received A779 (*P*group < 0.05) in non-2K1C rats. Such findings were not detected in 2K1C hypertensive rats. For example, in estradiol-treated rats that received A779, at 1000 ng/kg/min of Ang II infusion, RBF reduced from 1.6 ± 0.2 to 0.89 ± 0.19 ml/min in non-2K1C rats, and it reduced from 1.6 ± 0.2 to 1.2 ± 0.2 ml/min in 2K1C rats.

**Conclusion:**

Hypertension induced by 2K1C may attenuate the role of A779 and estradiol in renal vascular responses to Ang II infusion. Perhaps, this response can be explained by the reduction of Ang II type 1 receptor (AT1R) expression in the 2K1C hypertensive rats.

## 1. Introduction

High blood pressure caused about 9.4 million deaths and more than half of all strokes and ischemic heart diseases [1]. It is expected that more than one billion people worldwide will suffer from hypertension in 2025 [2]. Renal artery stenosis is responsible for hypertension in 2-4% of patients, and fibromuscular dysplasia and atherosclerosis obliterans are the two types of renovascular hypertension [3]. The renin-angiotensin system (RAS) [4] and estradiol [5] are two important items which regulate arterial blood pressure in women and need to be considered in hypertension condition.

Angiotensin (Ang) II insert its effects by two types of 1&2 receptors (AT1R and AT2R), while Ang 1-7 acts via Mas receptor (MasR) [6]. Heterodimerization and functional interactions also exist between MasR and AT1R or AT2R [7].

The RAS component expressions are different between the sexes [4], and females are less sensitive to Ang II-induced hypertension [8] due to the higher AT2R/AT1R ratio in females [9, 10]. Response to Ang II in the female is mediated by AT2R [9] through an estrogen-dependent mechanism [11]. Renal MasR expression is higher in female than male rats [12], and renal blood flow (RBF) response to Ang II infusion in normotensive female rats is higher than that of males [13]. MasR blockade [8] or angiotensin-converting enzyme 2 (ACE2) knockout [14] also reverse gender differences in response to Ang II infusion.

In experimental models of hypertension, intrarenal RAS modulation by sex hormones is involved in sexual dimorphism [15, 16]. It has been shown that the female rat is protected against renovascular hypertension due to intrarenal Ang 1-7-ACE2-MasR pathways [17, 18]. In hypertensive rats, renal cortical Ang 1-7 levels were significantly higher in females than males before and after exogenous Ang II infusion. Possibly, female hypertensive rats may convert most of the exogenous Ang II to Ang 1-7 [8].

Two kidneys-one clip (2K1C) provides a model of Ang II-induced hypertension. Kim et al. have shown unsuppressed expression of AT1R, while MasR expression decreased in the clipped kidney [19]. Also, five weeks after 2K1C hypertension induction, the AT1R/MasR ratio in the cortex of the clipped kidney is elevated [19].

Due to Ang II and Ang 1-7 receptor interaction [20–22], the direct effect of RAS on hypertension [23], and the effect of estradiol on RAS component regulation [15, 16], the renal vascular responses in the hypertensive animal may be different from that of the nonhypertensive one. Accordingly, we hypothesized that hypertension is a factor that may limit the effects of estrogen and MasR on the renal vascular responses to Ang II administration. To test this hypothesis, the ovariectomized rats simultaneously were subjected to 2K1C and estradiol therapy, and renal vascular responses to Ang II administration with and without MasR blockade (A779) were determined. The outcome may provide some information to understand the interaction between renovascular hypertension and RAS components in the clinic.

## 2. Materials and Methods

### 2.1. Animals

Forty-four female Wistar rats (7-9 weeks) were obtained from the Water and Electrolyte Research Center Animal House, Isfahan University of Medical Sciences. The rats were housed at a temperature of 23°C–25°C with a 12 h light/dark cycle, and they had free access to rat chow and tap water ad libitum. This animal experiment was approved by the Ethics Committee of the Isfahan University of Medical Sciences and followed the NIH guidelines for the treatment of animals (IR.MUI.MED.REC.1397.327). Animals underwent surgery for ovariectomy, and they were classified into non-2K1C (control or normotensive) and 2K1C (hypertensive).

### 2.2. Ovariectomy

The animals were anesthetized with chloral hydrate (450 mg/kg; IP) and xylazine (10 mg/kg; IP). Ovariectomy was performed by making a 2 cm incision in the subabdominal area, and the abdominal muscles were opened, and the intestine was retracted. Ovarian tubes were ligated to prevent bleeding, and the ovaries were removed carefully. The muscle and skin incisions were sutured, and the animals were placed under a heated lamp for recovery [24].

### 2.3. Two Kidneys-One Clip (2K1C) Hypertension

Simultaneously with ovarian resection, 21 animals among 44 were subjected to implement 2K1C by an incision on the right side of the abdomen, the right renal artery was isolated, and a U-shaped silver clip (lumen diameter of 0.2 mm) was placed around the artery to induce partial occlusion [17]. The other 23 ovariectomized animals also had a complete surgical intervention, just without a silver clip around the artery. These animals were considered control or normotensive (non-2K1C). After four weeks, we used the left kidney (nonclipped) for the measurements.

### 2.4. Estradiol Supplementation

22 animals selected from non-2K1C and 2K1C rats received high-dose estradiol valerate (Aburaihan Co., Tehran, Iran) dissolved in sesame oil via intramuscular injections for 4 weeks (500 *μ*g/kg/week, IM) [24]. The nonestradiol-treated groups (23 animals) from non-2K1C and 2K1C rats received sesame oil alone as the placebo. After 28 days, a surgical preparation was performed; the animals underwent surgery to record their blood pressure and determine the renal vascular response to Ang II infusion.

### 2.5. Experimental Design Groups

Based on the above information, 4 study groups were designed.

*Group 1 (n* = 12): the non-2K1C ovariectomized rats were treated with sesame oil as a placebo. The group was divided into two subgroups that received A779 (*n* = 6) or its vehicle (*n* = 6).

*Group 2 (n* = 11): the non-2K1C ovariectomized rats were treated with estradiol in sesame oil (500 *μ*g/kg/week). The group was divided into two subgroups, which received A779 (*n* = 5) or its vehicle (*n* = 6).

*Group 3 (n* = 11): the 2K1C ovariectomized rats were treated with sesame oil as a placebo. The group was divided into two subgroups that received A779 (*n* = 6) or its vehicle (*n* = 5).

*Group 4 (n* = 11): the 2K1C ovariectomized rats were treated with estradiol in sesame oil (500 *μ*g/kg/week). The group was divided into two subgroups that received A779 (*n* = 5) or its vehicle (*n* = 5).

### 2.6. Surgical Preparation

On the day of the experiment, the rats were anesthetized with urethane (1.7 g/kg^−1^ i.p.; Merck, Germany), and the trachea was cannulated to facilitate spontaneous breathing. The left jugular vein, left carotid, and left femoral arteries were catheterized by a polyethylene tube (PE 9658, Microtube Extrusions, North Rocks NSW, Australia) for drug infusion, systolic blood pressure (SBP) and mean arterial pressure (MAP), and renal perfusion pressure (RPP) measurements. To record the pressures, arterial catheters (carotid and femoral) were connected to a pressure transducer and a bridge amplifier (ADInstruments, Australia) and attached to a data acquisition system. The rats were placed in a lateral position, and the left kidney was exposed and gently separated from the surrounding tissues and placed inside the kidney cup. Then, its artery was isolated to place an ultrasound flow probe (TRANSONIC MA0.7PSB, Flow probe, USA) around it for direct RBF measurement. In addition, the abdominal aorta was isolated just above the renal artery, and an adjustable occluder was placed around the aorta to control RPP during Ang II infusion. The SBP, MAP, RPP, and RBF were measured continuously over the experiment.

### 2.7. Experimental Protocol

Following the surgical preparation, the rats were observed for 30 minutes to achieve a stabilization condition. MasR antagonist (A779) (Bachem Bioscience Inc., King of Prussia, PA, USA) was injected by a bolus dose (50 *μ*g/kg) and then was infused by a dose of 50 *μ*g/kg/h [13], and its vehicle (0.9% saline) in equal volume was administrated until the end of the experiment. The vehicle or antagonist is infused by using a microsyringe pump (New Era Pump System Inc., Farmingdale, NY, USA). 30 minutes post infusion of A779 or its vehicle infusion was considered a time for antagonist effect. Then, intravenous infusion of Ang II was commenced in a dose-related manner (30, 100, 300, and 1000 ng/kg/min) using a microsyringe pump (New Era Pump System Inc., Farmingdale, NY, USA). Each dose was administered for 15 minutes. The last 3-5 minutes of each stage was used for measurement of SBP, MAP, RPP, and RBF. Renal vascular resistance (RVR) was determined by the RPP/RBF ratio. Finally, rats were killed by an overdose of an anesthetic drug, and the kidneys and uterus were removed and weighed.

### 2.8. Statistical Analysis

Data were expressed as the mean ± SEM and were analyzed using the statistical software SPSS 20. One-way analysis of variance (ANOVA) was applied to baseline data. The effects of antagonist or its vehicle treatments on basal variables were compared via repeated-measures ANOVA with the factor group and treatment (before and during drug treatment) and their interaction. Post hoc analysis LSD was used to determine specific effects within each group. MAP, RPP, RBF, and RVR responses to Ang II are reported as a percent change from the baseline values and were compared via repeated-measures ANOVA with the factor group and treatment (30, 100, 300, and 1000 ng kg ^−1^ min^−1^ Ang II) and their interaction. *P* < 0.05 was considered statistically significant.

## 3. Results

### 3.1. Baseline Measurements

No significant differences were observed in basal SBP, MAP, RPP, and RBF normalized to left kidney weight (RBF/gLKW) during the equilibrium period before administration of A779 or its vehicle (Table 1). However, significant differences were detected in the SBP, MAP, and RPP between non-2K1C and 2K1C animals which confirmed the induction of hypertension in 2K1C rats. Also, uterus weight per 100 g body weight (UT/100gBW) was greater in estradiol-treated animals compared to vehicle-treated animals which confirmed the effect of estradiol (Table 1).

### 3.2. Effect of Antagonist

**Figure 1:**
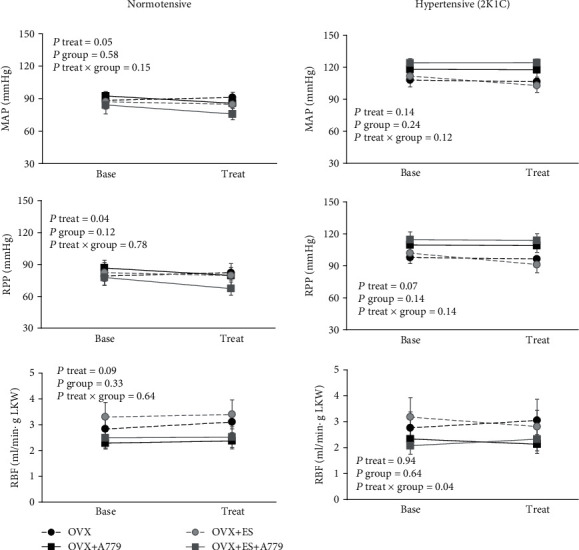
The antagonist or its vehicle effects: hemodynamic variables before and after administration of A779 or its vehicle in two models of normal and 2K1C ovariectomized rats treated with estradiol or its vehicle. Data are presented as the mean ± SEM. The *P* values were derived from repeated-measures ANOVA with factor treatment, group, and their interactions. MAP: mean arterial pressure (mmHg); RPP: renal perfusion pressure (mmHg); RBF/LKW: renal blood flow per gram left kidney weight (ml/min·g). OVX, ES, and A779 stand for ovariectomized rats, estradiol treatment, and Mas receptor antagonist administration, respectively.

Although there was a little change in MAP and RPP (*P*treat ≤ 0.05) responses to A779 or its vehicle infusion in non-2K1C rats, no difference was detected between the subgroups of A779 and its vehicle. Also, in 2K1C animals, the vehicle or antagonist did not alter MAP, RPP, and RBF/gLKW (Figure 1). For example, across 23 non-2K1C rats, MAP and RPP percentage (%) change decreased by 3.4 ± 1.9% and 4.3 ± 2.4%, respectively, and RBF increased by 4.9 ± 2.9%, while in 21 2K1C rats, MAP, RPP, and RBF decreased by 1.9 ± 1.6%, 2.8 ± 1.9%, and 1.6 ± 3.9%, respectively.

### 3.3. Responses to Ang II Infusion

**Figure 2:**
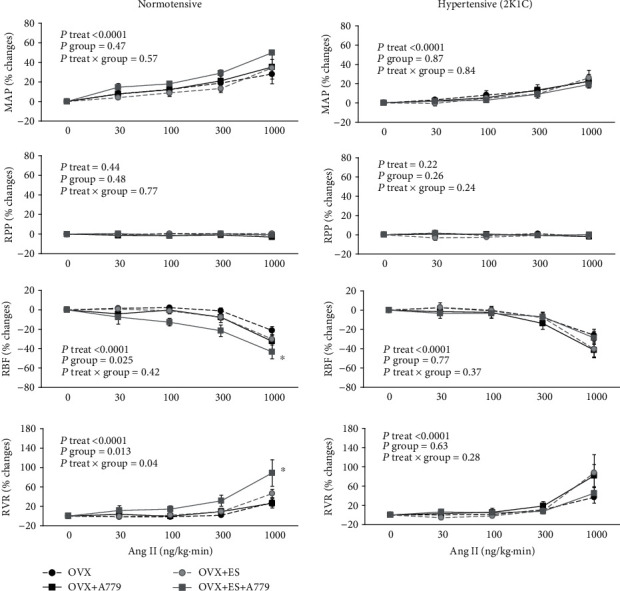
Effects of vehicle or A779 on responses to Ang II infusion in two models of normal and 2K1C ovariectomized rats. Data are shown as the mean ± SEM. The data presented as a percentage change from baseline. *P* value was derived from repeated-measures ANOVA with factors treat, group, and their interaction. ^∗^Significant difference from other subgroups (*P* < 0.05). OVX, ES, and A779 stand for ovariectomized rats, estradiol treatment, and Mas receptor antagonist administration, respectively.

Ang II infusion resulted in dose-related increases in MAP that were similar in the non-2K1C and 2K1C rats (*P*treat < 0.0001) (Figure 2). This increase was similar in the A779 or its vehicle subgroups, though it was greater in the non-2K1C than 2K1Cs. In all groups, RPP was kept relatively constant during graded Ang II infusion by manipulation of the aortic clamp around the abdominal aorta above the renal artery. Therefore, no significant difference in RPP was expected (Figure 2).

RBF % change decreased, and RVR % change increased in a dose-related manner in response to Ang II infusion in non-2K1C and 2K1C rats (Figure 2; *P*treat < 0.0001). However, in non-2K1C ovariectomized estradiol-treated rats, while A779 was significantly influenced, the RBF % response to Ang II infusion was increased significantly (*P*group < 0.05), which was different from other subgroups. On the other hand, in 2K1C ovariectomized estradiol rats, while A779 did not influence, the RBF response to Ang II infusion did not differ between the subgroups (Figure 2). For example, the dose of 1000 ng kg^−1^ min^−1^ Ang II reduced RBF from 1.6 ± 0.2 to 0.89 ± 0.19 ml/min and increased RVR from 46.3 ± 7.1 to 88.6 ± 20.2 mmHg·min/ml in ovariectomized non-2K1C estradiol-treated rats that received A779. While in 2K1C estradiol-treated rats that received A779, Ang II infusion (1000 ng kg^−1^ min^−1^) reduced RBF from 1.6 ± 0.2 to 1.2 ± 0.2 ml/min and increased RVR from 73.4 ± 8.8 to 109.3 ± 19.3 mmHg·min/ml (Figure 2).

## 4. Discussion

The main objective of this study was to determine the role of MasR and estradiol on renal hemodynamic responses to Ang II infusion in normotensive and 2K1C hypertensive rats. The major finding indicated that 2K1C-induced hypertension attenuated the effect of A779 and estradiol on renal hemodynamic responses to Ang II infusion.

Three points must be discussed here. First, there are some evidences that hypertension affects the RAS components in experimental hypertension models [17, 19, 25]. AT1R expression reduces in both kidneys, and in contrast, MasR expression decreases in the clipped kidney of 2K1C rats, while 5 weeks after clipping, the AT1R/MasR ratio in the cortex of the clipped kidney elevates [19]. In addition, AT1aR protein decreases in both kidneys in 2K1C hypertensive rats [24]. Therefore, AT1R expression reduction in the nonclipped kidney in the 2K1C model may provide a lower response of RBF and RVR to Ang II administration.

Second, the dose of estradiol is another point that affects RAS components. The vasodilatory effect of estradiol is exerted by AT2R, but a higher AT_1_R/AT_2_R ratio was found in female hypertensive animals [10]. However, a previous report indicated that a high dose of estradiol enhanced RBF and RVR responses to Ang II administration in normotensive ovariectomized rats [24]. During the development of Ang II-induced hypertension, about one-fifth of vascular contraction occurs through the AT_2_R [26], and a high dose of estradiol caused a contraction in vessels [24]. On the other hand, high levels of estrogen during pregnancy or the use of oral contraceptives stimulates the RAS and may lead to a significant increase in plasma renin levels [27]. Ovariectomy itself has been shown to increase AT_1_R expression and binding in the kidney, heart, and brain [28]. Collectively, a high dose of estradiol is a factor to increase vascular contractile response to Ang II administration [24].

Third, it seems that MasR plays an important role in the 2K1C hypertension model. MasR is a sex-specific receptor, and its blockade reduces RBF in female, but not in male, rats [13]. Acute intrarenal MasR blockade did not alter renal function in normotensive rats, but it caused a significant reduction of renal hemodynamics in 2K1C rats [29]. Lee et al. reported that female rats were more resistant to hypertension than males due to increased intratubular levels of ACE2, MasR, and Ang 1-7 [17], and A779 caused an increase in blood pressure [30]. Ang II infusion decreased AT1R and increased AT2R expression only in hypertensive male rats while MasR expression increased only in females [8]. Furthermore, MasR expression did not change in the kidney of hypertensive rats [31]. It also is demonstrated that ACE2 and MasR expression reduced in the cortex and medulla of both kidneys in the Goldblatt renovascular model [32, 33]. Accordingly, the decrease of RBF and RVR responses to Ang II infusion in 2K1C ovariectomized estradiol-treated rats when MasR was blocked may be related to the reduction of AT1R expression [19].

This study involved some limitations that should be explained here. We did not measure the expression of MasR, AT1R, and AT2R that are possibly altered by estradiol therapy and 2K1C-induced hypertension. We also did not use different doses of estradiol to find the dose-response in renal hemodynamic responses to Ang II administration. However, the increased uterus weight in estradiol-treated animals was considered evidence for the effectiveness of estradiol therapy.

## 5. Conclusion

Hypertension is a phenomenon that influenced RAS components. So, hypertension induced by 2K1C attenuated the effect of A779 and estradiol in RBF and RVR responses to Ang II infusion due to reduction of expression of AT1R and MasR in the nonclipped kidney. These findings may be considered in the clinic for patients with renovascular hypertension such as renal artery stenosis.

## Figures and Tables

**Table 1:** Baseline data for SBP (mmHg), MAP (mmHg), RPP (mmHg), RBF/gLKW (ml/min·g), and UT/100gBW in two models of normal and 2K1C ovariectomized rats.

Model	Non-2K1C (control, normotensive)						2K1C (hypertensive)					
Subgroup	*n*	SBP	MAP	RPP	RBF/gLKW	UT/100 gBW	*n*	SBP	MAP	RPP	RBF/gLKW	UT/100 gBW
OVX	6	118 ± 7.6	88.4 ± 4.9	79.1 ± 5.0	2.84 ± 0.42	0.02 ± 0.003	5	145.4 ± 3.8^#^	108.0 ± 6.4^#^	97.8 ± 5.6^#^	2.76 ± 0.61	0.025 ± 0.005
OVX+A779	6	111.5 ± 6.4	92.5 ± 3.9	86.7 ± 5.2	2.28 ± 0.23	0.02 ± 0.004	6	149.4 ± 5.8^#^	118.0 ± 7.4^#^	109.6 ± 7.2^#^	2.33 ± 0.34	0.026 ± 0.003
OVX+ES	6	127.4 ± 4.7	87.2 ± 6.8	82.3 ± 11.8	3.3 ± 0.56	0.09 ± 0.004^∗^	5	150.7 ± 2.7^#^	111.7 ± 3.9^#^	101.9 ± 4.9^#^	3.18 ± 0.75	0.102 ± 0.008^∗^
OVX+ES+A779	5	115.6 ± 3.9	84.2 ± 8.2	77.7 ± 7.5	2.5 ± 0.39	0.11 ± 0.008^∗^	5	157.7 ± 2.4^#^	124.1 ± 4.2^#^	114.6 ± 7.2^#^	2.10 ± 0.33	0.099 ± 0.013^∗^
*P*_ANOVA_	—	0.30	0.81	0.86	0.36	<0.0001	—	0.37	0.29	0.31	0.54	<0.0001

Data are presented as the mean ± SEM. The *P*values were derived from one-way ANOVA. Specific contrasts were generated by LSD post hoc comparisons. OVX: ovariectomized; ES: estradiol; LKW: left kidney weight; BW: body weight; UW: uterus weight; KW: kidney weight; SBP: systolic blood pressure; MAP: mean arterial pressure; RPP: renal perfusion pressure; RBF: renal blood flow per gram kidney weight; RVR: renal vascular resistance. ^∗^Significant difference from nonestradiol groups (*P* < 0.05). ^#^Significant difference from non-2K1C groups (*P* < 0.05).

## Data Availability

Data will be available on request.
